# Clinical, Radiological, Microbiological, and Histopathological Aspects of Acquired Dacryocystoceles

**DOI:** 10.1155/2014/396782

**Published:** 2014-06-11

**Authors:** Selam Yekta Sendul, Sonmez Cinar, Halil Hüseyin Çağatay, Mehmet Demir, Burcu Dirim, Dilek Guven

**Affiliations:** ^1^Department of Ophthalmology, Sisli Etfal Training and Research Hospital, Halaskargazi Street, Etfal Home Street, Şişli, 34371 İstanbul, Turkey; ^2^Department of Ophthalmology, Faculty of Medicine, Kafkas University, Pasacayiri Street, 36301 Kars, Turkey

## Abstract

*Purpose*. The aim of this study is to investigate the etiology and the clinical, microbiological, histopathological, and radiological findings of acquired dacryocystoceles. *Methods*. In this retrospective study, we reviewed the clinical records of 10 eyes of 8 patients with dacryocystoceles who underwent external dacryocystorhinostomy (DCR) surgery. Etiology, presenting symptoms and radiological findings as well as microbiological and histopathological assessment results and outcome were analyzed. *Results*. The records of 8 patients with dacryocystoceles were included in this study. In the histopathological evaluations of the samples collected from the lacrimal sac wall, chronic inflammation was found in all biopsied samples and fibrosis was observed in two histopathological evaluations. Computerized tomography (CT) imaging showed fluid collection separated from adjacent tissues by a thin rim, corresponding to dacryocystoceles in the sac. In the microbiological culture examination of samples collected from the fluid within the cyst, no bacterial growth in 5 eyes, gram-negative bacillus growth in 3 eyes, and gram-positive cocci growth in 2 eyes were found. *Conclusions*. Acquired dacryocystoceles were observed extremely rarely and a definite pathogenic agent could not be identified in any of the cases, either microbiologically or histologically, whereas chronic inflammation was detected in all cases in our study.

## 1. Introduction

An acquired dacryocystocele is a diffuse and centrifugal enlargement of the lacrimal sac that forms as a result of obstruction of lacrimal drainage system proximally at the level of the common canaliculus and distally at the level of nasolacrimal duct [1]. It is characterized by swelling in the medial canthal region accompanied by watery eyes. It is common in children yet rare in adults. Findings associated with dacryocystocele are irreversible fibrotic narrowing in the lacrimal system, osseous changes in the nasolacrimal duct and chronic and low-grade inflammation [1]. Adult dacryocystoceles are often observed after dacryocystitis; however, they may also develop idiopathically or due to autoimmune fibrous diseases, sinusitis and paranasal sinus mucoceles, after trauma or osseous configuration in the dysplastic duct wall and tumors, or secondarily to dacryocystorhinostomy surgeries. In computerized tomography (CT) and magnetic resonance imaging (MRI), a dacryocystocele appears as a fluid collection that is separated from other solid structures by a thin rim [2]. Differential diagnosis includes malformations of the lacrimal sac, diverticulum, dermoid and epidermoid cysts, encephalocele, primary epithelial tumors of the sac, and external tumors [1].

In this study, we aimed to determine the probable etiology as well as clinical, microbiological, histopathological, and radiological findings of dacryocystoceles in acquired dacryocystocele cases. To our knowledge, this is one of the largest case series published of acquired dacryocystoceles in adult patients.

## 2. Material and Method

In the study, the charts of patients who had undergone external dacryocystorhinostomy (EDCR) surgery in our clinic between 2008 and 2013 were reviewed retrospectively, and 10 eyes of 8 patients who were diagnosed with dacryocystocele were included in the study. Two (25%) out of 8 patients had bilateral dacryocystoceles. The median follow-up time for those patients was 22 months (range 13 to 38 months). The study was conducted in accordance with the tenets of the Declaration of Helsinki by obtaining written consent from all patients, with the approval of the local ethical review board. Complete ophthalmologic examinations, nose examinations, and CT imaging were done preoperatively for all cases. Those patients who had epiphora complaints, who were observed with a palpable mass at the medial chantal region and no pus coming out from the lower and upper punctum when the mass was pressed with a finger, and who showed a soft ending when a lavage cannula was placed into the punctum were diagnosed with dacryocystocele. All patients underwent external DCR surgery with silicone tube placement. Culture specimens were collected from the surgically excised lacrimal sac walls and fluid samples were collected perioperatively for microbiological analysis. The material was also smeared onto sterile labeled glass slides for 10% potassium hydroxide wet mount, Gram stain, Giemsa stain, Ziehl-Neelsen acid fast stain, and Kinyoun's acid fast stain. Specimens were inoculated into sheep's blood agar, chocolate agar, Sabouraud's dextrose agar, brain heart infusion broth, and thioglycollate medium. At the stage of preparation of preoperative lacrimal sac flaps, incisional biopsy samples were collected from the lacrimal sac for histopathological assessment to establish a differential diagnosis. Histological examination was performed using haematoxylin and eosin staining of sections. During lacrimal intubation, an obstruction was experienced in the common canaliculus and it was noted that the obstruction was overcome with a click sound while inserting the intubation tube. Routine external DCR operations were completed and control examinations were made on postoperative day 1, week 1, and month 1 and month 3, month 6, and then yearly. Anatomic success was assessed by diagnostic probing with a hard stop and irrigation through the passage in all adult patients. Absence of epiphora was defined as functional success.

## 3. Results

**Figure 1:**
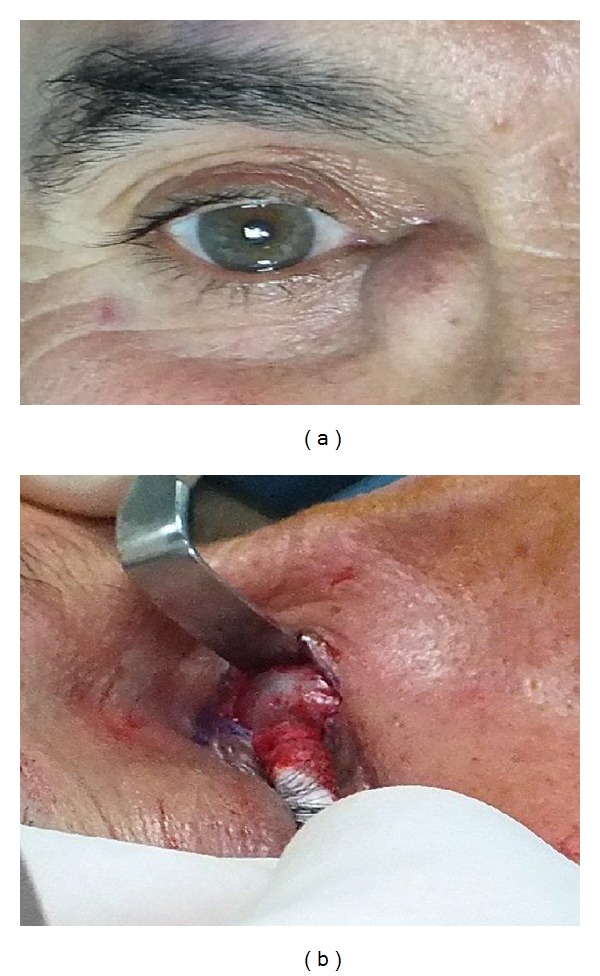
(a) 54-year-old male patient. In the right eye lacrimal sac region, a solid, immobile mass is observed, pressure on which does not cause pus discharge. (b) Perioperative image of the lesion. It is observed that the lacrimal sac is quite big and has a bluish color.

**Figure 2:**
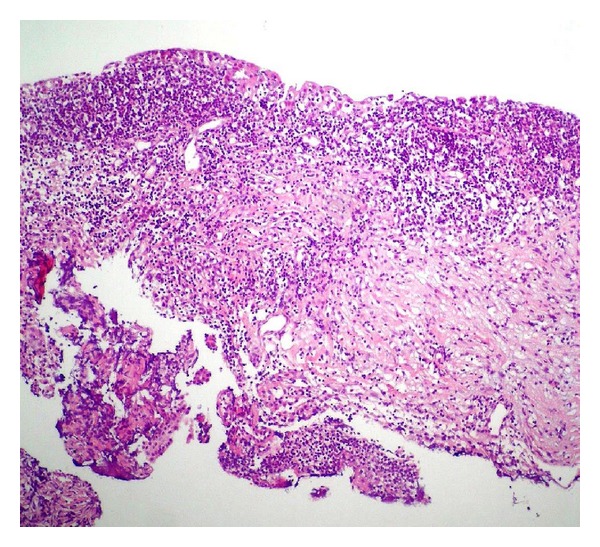
A portion of the lacrimal sac biopsy material which is taken from 35-year-old female patient. Epithelial damage together with common lymphohistiocytic infiltration in the subepithelial region is observed (hematoxylin and eosin, magnification ×200).

**Figure 3:**
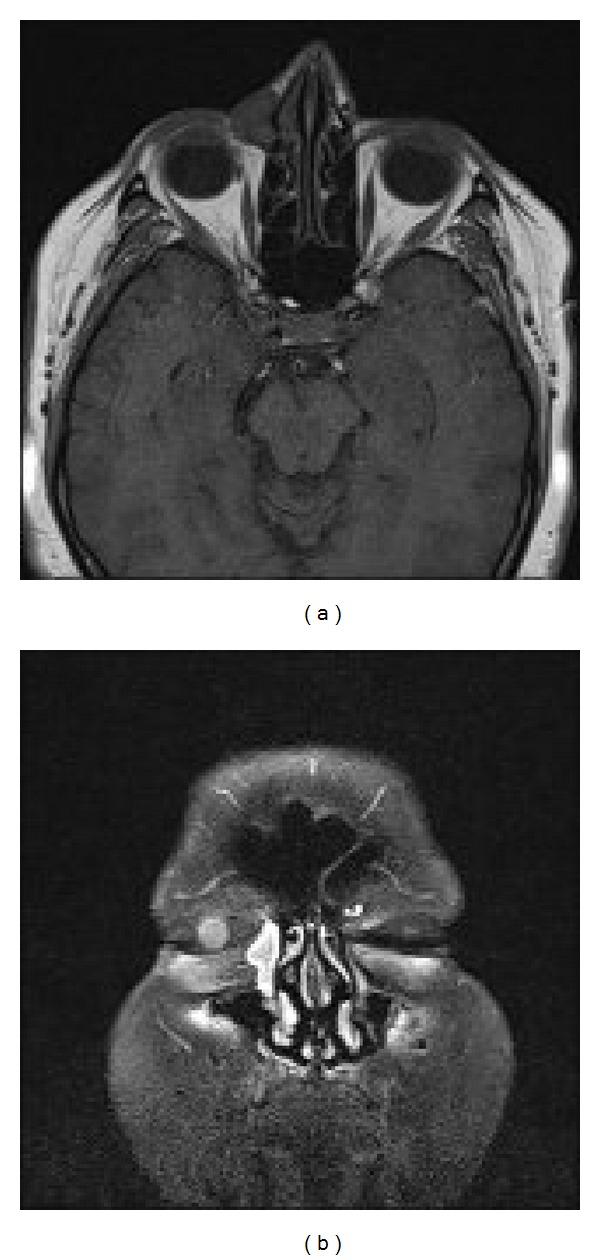
In MR imaging with axial and coronal contrast, it is observed that the right lacrimal sac is quite a lot bigger compared to the left eye and differentiates from the adjacent tissues by way of contrast involvement.

The records of 412 patients who underwent external DCR surgery due to acquired nasolacrimal duct obstruction were reviewed retrospectively. A total of 10 eyes of 8 patients (4 females and 4 males, aged 29 to 75, mean 52 ± 16.1 years) with dacryocystoceles were included in this study. The most common initial symptoms and findings were epiphora (100%) and palpable mass (100%) in the medial canthal area (Figures 1(a) and 1(b)). In the histories of the patients, it was observed that there was the history of a mass in the lacrimal sac region unaccompanied by inflammation in 7 eyes (70%) and dacryocystitis history in 3 eyes (30%) which had been treated in accordance with the diagnosis of acute dacryocystitis. In the initial examination there was no acute dacryocystitis, trauma history, or any local or systemic disorder which could result in damage in the lacrimal system. In nose examinations, no peculiarities were observed in none of the cases (Table 1). Computerized tomography imaging demonstrated the fluid collection separated from adjacent tissues by a thin rim corresponding to a dacryocystocele in the sac, but no further lesions (Figures 3(a) and 3(b)). In the histopathological evaluation of the sample collected from the lacrimal sac wall during surgery, chronic inflammation findings were observed in all eyes (100%) as well as fibrosis in 2 eyes (20%) (Figure 2). Again, in the microbiological examination of the fluid samples collected from within the cyst during surgery, no bacteria growth in 5 eyes (50%), gram negative bacillus in 3 eyes (30%), and gram positive cocci in 2 eyes (20%) were observed (Table 1). All cases were treated with external DCR combined with bicanalicular silicone tube placement, and no complications were observed, at least at annual follow-ups. Anatomical and functional success was achieved in all cases (100%).

## 4. Discussion

An acquired dacryocystocele is a rare, benign, and painless mass located at the side of the lacrimal sac. Dacryocystoceles usually occur congenitally or less frequently as a result of chronic inflammation, surgeries, and tumors which originate from medial canthal region or sinuses, radiation therapy, or as a side effect of chemotherapeutic drugs [2]. Knowledge of the pathogenic mechanism of acquired dacryocystoceles is not clear. Previously published literature about dacryocystoceles is mostly related to the congenital form. In congenital cases particularly, dacryocystocele occurrence has been explained as the obstruction of the common canaliculi and lacrimal sac junction, by enlargement of the sac fundus which limits the movement of the Rosenmuller valve indirectly, and obstruction of the distal duct by the presence of a persistent membrane between the lacrimal duct and nasal mucosal epithelium, which is present in many newborns [3–5].

In patients who develop dacryocystitis following nasolacrimal duct obstruction (NLDO), infective materials that accumulate in the sac may enlarge the sac in a form similar to congenital dacryocystocele [3]. Reasons such as the limitation of the movement of the valve, damage to the valve epithelium due to an infective environment, and chronic inflammation may lead to irreversible synechia in the common canaliculus [2]. In our study, a history of dacryocystitis was detected in 3 eyes (30%), whereas there was no history of dacryocystitis in 7 eyes (70%); therefore, these cases were assessed as idiopathic dacryocystocele. Then, one comes to the question as to why the common canaliculus does not get blocked after every chronic dacryocystitis, or in other words, why dacryocystocele does not develop in all chronic dacryocystitis cases. Taking this question as the starting point, we strived to understand the histopathological causes for patients who develop dacryocystocele after NLDO, by collecting preoperative microbiological samples and perioperative biopsy samples from the lacrimal sac. Histopathologically, we detected inflammation in all patients. Similarly, in a case report, Lai et al. histologically evaluated dacryocystoceles and detected chronic inflammation from the cystic wall of the patients' samples, [6] whereas Woo and Kim, in their series of 4 cases, identified partial necrotic changes in addition to chronic inflammation [7]. In our study, we collected specimens from all patients with dacryocystoceles perioperatively, and only the cultures of 5 eyes reproduced bacteria. Further, as the bacteria strains produced were different from each other, we concluded that the formation of dacryocystoceles is not associated with a specific pathogenic agent. Perry et al. reported that* Prevotella* (gram (−) bacillus) and* Peptostreptococcus* (an aerobic gram (+) coccus) grew in the microbiological analysis of specimens from the dacryocystomucopyocele fluid, which are members of the normal otolaryngeal flora [1]. Koltsidopoulos et al. could not ascertain any etiology in their cases and defined them as idiopathic dacryocystocele [8]. Plaza et al. reported two familial cases one of which was determined as bilateral dacryocystoceles and the other as unilateral dacryocystoceles [9]. Woo and Kim reported bilateral dacryocystoceles in one out of the four cases which was a congenital case associated with punctual agenesis [7]. We observed bilateral dacryocystoceles in two cases none of which was associated with punctual or canalicular agenesis. This was another interesting finding.

Debnam et al. studied the imaging characteristics of dacryocystoceles that occurred after surgery for sinonasal cancers and reported that CT and MR imaging revealed characteristic findings such as a cystic, fluid filled structure with thin rim enhancement and no solid components [2]. In accordance with these findings, we performed CT imaging in all cases in our study, which demonstrated fluid collection separated from adjacent tissues by a thin rim corresponding to a dacryocystocele in the medial canthal area but no other pathologic lesions.

The treatment of dacryocystoceles includes conservative management and drainage followed by external or endoscopic DCR, combined with or without silicone tube placement or dacryocystectomy [7–10]. In the previous literature, it seems that mostly conservative management was preferred in congenital cases and dacryocystectomy was reserved for patients with dry eyes or those who were poor surgical candidates [1]. We preferred external DCR with silicone tube placement and found that it is a well tolerated and effective method that yields satisfactory results. Particularly when there is no upper lacrimal system absence, such as punctual or canalicular agenesis, external or endoscopic DCR can be the first choice of surgical technique. If there is an upper lachrymal system damage, then conjunctival DCR may be the first choice [7].

In conclusion, dacryocystocele should be taken into consideration in the diagnosis of any painless cystic mass in the lacrimal sac area, and CT imaging can be helpful for diagnosis, whereas surgical interventions combining silicon tube placement may give successful results. We found that although it is not possible to obtain a definite pathogenic agent in all cases of microbiological analysis of dacryocystocele specimens, dacryocystoceles may occur due to chronic dacryocystitis. It may also develop idiopathically or due to autoimmune fibrous diseases, sinusitis and sinus mucoceles, trauma or osseous configuration in the dysplastic duct wall, from tumors, and secondarily to dacryocystorhinostomy. Previous studies about adult dacryocystoceles are mostly case reports or case series. To our knowledge, however, this study is one of the largest series; therefore we think that adult dacryocystoceles are more common than expressed through published literature. Further studies on adult dacryocystoceles with larger sample sizes are required to determine the pathologic mechanism and the gold standard treatment modalities.

## Figures and Tables

**Table 1:** Clinical characteristics and microbiological culture results of the 8 patients with dacryocystoceles (the term “idiopathic” is used for cases which do not have a history of inflammation and the term “chronic dacryocystitis” is used for cases which underwent medical treatment for acute dacryocystitis).

Case	Age	Sex	Side	History	Follow-up time (month)	Microbiology
1	29	F	Bilateral/right	Chronic dacryocystitis	14	Strain 1: *Pantoea agglomerans* Starin 2: *Acinetobacter lwoffii*
			Bilateral/left	Chronic dacryocystitis	14	Strain 1: *Pantoea agglomerans* Starin 2: *Acinetobacter lwoffii*

2	54	M	Bilateral/right	Idiopathic	18	Methicillin resistant coagulase (−) Staphylococci
			Bilateral/left	Idiopathic	18	Methicillin resistant coagulase (−) *Staphylococci *

3	58	F	Right	Idiopathic	17	No bacteria growth

4	35	F	Left	Idiopathic	13	No bacteria growth

5	75	M	Right	Idiopathic	24	No bacteria growth

6	56	M	Left	Idiopathic	38	*Achromobacter* species

7	51	M	Left	Chronic dacryocystitis	30	No bacteria growth

8	58	F	Right	Idiopathic	22	No bacteria growth
